# Electronic-Nose Applications for Fruit Identification, Ripeness and Quality Grading

**DOI:** 10.3390/s150100899

**Published:** 2015-01-06

**Authors:** Manuela Baietto, Alphus D. Wilson

**Affiliations:** 1 Dipartimento di Scienze Agrarie e Ambientali-Produzione, Territorio, Agroenergia (DISAA), Università degli Studi di Milano, via Celoria 2-20133 Milano, Italy; 2 USDA Forest Service, Center for Bottomland Hardwoods Research, Southern Hardwoods Laboratory, 432 Stoneville Road, Stoneville, MS 38776-0227, USA; E-Mail: dwilson02@fs.fed.us

**Keywords:** electronic aroma detection, e-nose, fruit volatiles, volatile organic compounds

## Abstract

Fruits produce a wide range of volatile organic compounds that impart their characteristically distinct aromas and contribute to unique flavor characteristics. Fruit aroma and flavor characteristics are of key importance in determining consumer acceptance in commercial fruit markets based on individual preference. Fruit producers, suppliers and retailers traditionally utilize and rely on human testers or panels to evaluate fruit quality and aroma characters for assessing fruit salability in fresh markets. We explore the current and potential utilization of electronic-nose devices (with specialized sensor arrays), instruments that are very effective in discriminating complex mixtures of fruit volatiles, as new effective tools for more efficient fruit aroma analyses to replace conventional expensive methods used in fruit aroma assessments. We review the chemical nature of fruit volatiles during all stages of the agro-fruit production process, describe some of the more important applications that electronic nose (e-nose) technologies have provided for fruit aroma characterizations, and summarize recent research providing e-nose data on the effectiveness of these specialized gas-sensing instruments for fruit identifications, cultivar discriminations, ripeness assessments and fruit grading for assuring fruit quality in commercial markets.

## 1. Introduction

Fruit quality is judged by consumers primarily from their perception of the acceptability of fruits based on characteristics including visual appeal (lack of blemishes, color, size, and texture), ripeness, aroma and flavor. The quality of fruits (as measured by aroma, flavor, color, and textural characteristics) constantly changes during fruit development from pre-harvest through post-harvest stages as fruits grow and ripen, and during maintenance in storage [1]. Personal consumer preferences for different types of fruits are reflected in their particular choices of fruit varieties or cultivars selected for purchase. Fruit varieties vary widely in aroma characteristics due to differences in the composition of aromatic volatiles present in fruit aromas which are ultimately determined by plant genetics [2,3]. Previously, professional human graders and panelists have been used to judge fruit quality based on visual and aroma characteristics for selecting and evaluating fruits for ripeness at harvest and salability in commercial fruit markets [4]. The advent of electronic-nose (e-nose) devices has offered new alternative tools for grading fruits and other perishable foods using more consistent qualitative and quantitative measures of aroma characteristics that avoid the highly variable subjective opinions of human graders [5,6]. These instruments provide new means for characterizing fruit aromas for numerous applications ranging from the development of new fruit varieties by geneticists or fruit breeders to the timing of fruit harvests, transportation, storage operations (handling), and final selection by commercial dealers and retailers in fresh produce markets.

Fruit aroma is often the most valued characteristic determining fruit quality and consumer choice because aroma is usually the best indicator of fruit flavor. Electronic-noses are ideal digital, electronic devices for identifying, characterizing and grading fruit aromas from different fruits and fruit varieties because these instruments are capable of rapidly and consistently evaluating complex volatile gaseous mixtures without having to identify all of the chemical constituents present in the bouquet of fruit aromas [5,7]. E-noses contain a sensor array that evaluates all of the chemical constituents present in an aroma mixture (as a whole sample) and coverts the electronic output signals (via a transducer) from all of the sensors in the array and collectively assembles them to form a distinct digital pattern, sometimes referred to as an Electronic Aroma Signature Pattern (EASP) that is highly unique and specific to the particular gas mixture being analyzed [8,9]. In this way, the instrument output generates an aroma signature or smell-print that can be used to identify the particular type and variety of fruit being analyzed.

Fruits produce and release a wide variety of Volatile Organic Compounds (VOCs) that make up their characteristic aromas with esters, terpenoids, lactones and derivatives of amino acids, fatty-acids and phenolic compounds being the dominant classes of organic volatiles represented in fruit aromas [3]. Even though different fruits share some aromatic characteristics, each fruit has a distinctive aroma that depends upon the specific combination of VOCs present in the aroma mixture [10]. Whereas some specific volatiles are common to different fruit types, other fruit volatiles are specific to only one or only a few related fruits. Production and emission of volatiles from fruits is markedly influenced by numerous factors that interact in complex ways to determine fruit volatile composition. Multiple biochemical pathways are responsible for determining the final composition of volatile compounds released from different fruit types.

The purpose of this review is to summarize some of main chemical characteristics of fruit volatile gaseous mixtures which are conducive to characterization and analysis by electronic-nose technologies, to describe the diverse potential applications of e-nose technologies in the agro-fruit production sector of the agricultural production industry, and to provide examples of research that have demonstrated many ways in which e-nose devices have been utilized to distinguish between the fruit volatiles of different plant species and varieties for the purpose of analyzing and grading fruit quality and aroma characteristics.

## 2. Chemical Characteristics of Fruit Volatiles

Fruit aromas consist of a complex mixture of VOCs whose composition is specific to plant species and fruit variety [2,3]. Although different fruits often share many aromatic characteristics, each fruit has a distinctive aroma that depends upon the combination of volatiles, the concentration and the perception threshold of individual volatile compounds [10]. The most important aroma compounds include amino acid-derived compounds, lipid-derived compounds, phenolic derivatives, and mono- and sesquiterpenes [3]. Although fruit aromas are generally complex mixtures of a wide range of compounds, volatile esters often represent the major components of aroma volatiles present in rosaceous fruits such as apple (*Malaus domestica* Borkh.) and peach (*Prunus persica* L.) [11,12].

Fruit volatiles are mainly composed of VOCs in relatively few chemical classes, including primarily aliphatic esters, alcohols, aldehydes, ketones, lactones, terpenoids (monoterpenes, sesquitepenes) and apocarotenoids [13]. However, there are many thousands of volatile compounds represented by these chemical classes that make up the complex aroma mixtures of the numerous fruit types cultivated for agronomic markets of the world. Some of the predominant VOC principal components, comprising the distinctive aroma mixtures of selective fruit types which are representatives of the most common chemical classes found in fruit volatiles, are presented in Table 1. Volatile compounds in the aliphatic ester chemical class are the most abundant types of organic compounds found in many fruit volatiles; and esters are most responsible for the sweet smell of flowers and fruits of most angiosperms or seed plants [14]. In aromatic melon varieties for example, volatile esters predominate in fruit aromas that also contain aldehydes, short-chain alcohols, sesquiterpenes, norisoprenes, and aromatic sulfur-containing compounds [15]. Non-aromatic fruit varieties often have much lower levels of total volatiles and lack volatile esters [16]. Esters are a particularly important component of strawberry fruit aroma, accounting for 90% of the total number of volatiles in ripe strawberry fruit [17]. Esters also are the key volatiles responsible for the flavor characteristics of citrus [18].

Fruit volatiles, in addition to chemical class categorizations, may be classified as primary compounds (present in intact fruit tissue) or secondary compounds (produced as a result of tissue disruption of fruit tissue) [13]. Consequently, the condition of the fruit tissue being analyzed, either intact or disrupted, will influence the characteristics (chemical composition) and output patterns of the resulting aroma profiles. Some aroma compounds may be released only from cell disruption due to physical damage or injury to the fruit. Other fruit volatiles are more bound internally in the fruit, perhaps due to lower volatility or lack of direct exposure to the air as a result of fruit tissue compartmentalization.

Numerous factors affect fruit volatile chemical composition during all phases of the agronomic production process, including plant genetics, harvest time, fruit maturity, and agronomic environmental conditions, as well as postharvest handling, transportation and storage. Plant genetics, plant hormones, and environmental factors strongly influence the biosynthesis pathways responsible for the release of volatile aroma compounds from fruits under various conditions over time. The availability of primary precursor substrates for biosynthetic pathways producing aroma compounds is highly regulated both in amount and composition during fruit development [34]. All of these factors have varying effects on the aroma volatiles released at different stages of fruit development and after fruit harvest when the detached fruit is no longer influenced by the biochemical processes of the plant.

Fatty acids are the major primary precursor substrates of many aroma volatiles in most fruit types [2]. Aliphatic alcohols, aldehydes, ketones, organic acids, esters and lactones, ranging from C_1_ to C_20_, are all derived from fatty acid precursors through three key biosynthetic processes: α-oxidation, β-oxidation and the lipoxygenase pathway [35]. Volatiles derived from fatty acid precursors are important character-impact aroma compounds responsible for fresh fruit flavors at high concentrations.

The terpenoids comprise the largest class of plant secondary metabolites (about 20,000 identified), derived from the universal C_5_ precursor isopentenyl diphosphate (IPP) and its allylic isomer dimethylallyl diphosphate (DMAPP) from two independent pathways, the mevalonic acid (MVA) and methylerythritol phosphate (MEP) biochemical pathways, with many volatile products represented [36,37]. Terpenes are classified into monoterpenes, diterpenes and sesquiterpenes, depending on the number of repeating units of a 5-carbon molecule (isoprene), the structural unit of all terpenoids, present in the molecule. Hemiterpenes (C_5_), monoterpenes (C_10_), sesquiterpenes (C_15_), homoterpenes (C_11_ and C_16_), and some diterpenes (C_20_) are quite volatile VOCs because they have a high vapor pressure, allowing their rapid release into the atmosphere [13].

The complex gaseous mixtures of VOCs released from various fruit types (plant species), detectable by e-nose instruments, depend on the extent to which different metabolic pathways predominate in the generation of fruit volatiles as determined by genetic and environmental factors. Certain types of VOCs are more frequently associated with specific fruits as a result of unique combinations of metabolic pathways that control primary and secondary metabolite production in fruit tissues. The major chemical classes and representative VOCs found in fruit volatile mixtures, most associated as principal components derived from specific fruit types, are presented in Table 2. These associations do not preclude the occurrence of VOCs from many other chemical classes in volatile mixtures from each fruit type, but occur in different relative proportions (molar ratios) as lesser components in VOC mixtures derived from various fruit types.

The total number of aromatic compounds that contribute to fruit aromas vary considerably in different fruit types, but the complex mixture of VOCs found in individual fruit aromas usually is an extensive list of volatiles from different classes of organic compounds. Fruit aromas from fresh apples (*Malus domestica* Borkr) have been reported to contain at least 300 volatile compounds [51]. The total number and concentration of VOCs emitted by ripening apples are cultivar specific [52].

The epidermal tissue (peel) of apples produces a greater quantity of volatile compounds than internal fleshy (pericarp) tissues [53]. This higher capacity for aroma production by peel tissue is due to either the abundance of fatty acid substrates or higher metabolic activity in the peel [54,55]. Peach (*Prunus persica* (L.) Batsch) fruit aromas consist of about 100 volatile compounds, among them, C6 aldehydes and alcohols provide the green-note aroma, while lactones and esters are responsible for fruity aromas [56–58]. Esters, including hexyl acetate and (*Z*)-3-hexenyl acetate, are key odorants influencing the flavor characteristics of peach fruit [59]. Changes in these volatiles occur during fruit development and postharvest ripening [60]. Aldehydes tend to decline, while esters increase in the fruit during development. Postharvest treatments, low temperature and controlled atmosphere, can influence changes in peach aroma quality [12,61]. More than 300 VOCs have been identified in pear fruit (*Pyrus pyrifolia* Nak.) [62]. Methyl and hexyl esters of decadienoate are the main character-imparting compounds of European pear [62,63].

Apricot (*Prunus armeniaca* L.) fruit aromas have more than 200 different volatile compounds [64]. The most abundant volatile compounds by concentration were aldehydes, primarily hexanal and (*E*)-2-hexenal, that decreased in concentration during ripening [21,22].

Banana (*Musa* spp.) fruit aroma has about 250 VOCs [40], although the characteristic banana fruity top notes are from volatile esters, such as isoamyl acetate and isobutyl acetate that tend to increase in concentration during ripening [23,40]. Volatile compounds in citrus fruits accumulate in oil glands of flavedo and in the oil bodies of the juice sacs from which 100 VOCs have been identified, but varietal differences in the volatile profiles are primarily quantitative and only a few compounds are variety-specific [65–68].

Approximately 42 volatiles has been associated with the fruit aromas of southern highbush blueberry (*Vaccinium* species) cultivars [43]. Certain varieties contain a large amount of esters and C_6_ aldehydes, whereas others produce more terpenoids and less esters.

Melon (*Cucumis* and *Citrullus* species) fruit aromas have more than 240 VOCs identified in different varieties [69]. Melon fruits release numerous compounds, particularly C_9_ aliphatic compounds that are the major determinants of fruit quality as perceived by consumers. These compounds are strongly dependent on variety and particular physiological characteristics of the fruit. For example, climacteric melons (cantaloupes) have greater aroma intensity and shorter shelf life than less climacteric melons (honeydew melons) [70]. Volatiles derived from amino acids are major contributors to the aromas of both aromatic and non-aromatic melon varieties [71,72].

Grape (*Vitis vinifera* L.) fruit aromas contain many VOCs, including monoterpenes, C_13_ norisoprenoids, alcohols, esters and carbonyl compounds [73]. Grape varieties may be divided into aromatic and nonaromatic categories. Terpenoids are major volatiles in both red and white grapes [44]. At veraison, terpene production (in both Riesling and Cabernet Sauvignon varieties) generally is low, but Riesling grapes produced some terpenes (geraniol and α-muurolene) post-veraison.

Kiwi (*Actinidia* species) fruit aromas consist of more than 80 compounds with the major volatile components being methyl and ethyl butanoate, (*Z*)- and (*E*)-2-hexenal, hexanal, (*Z*)- and (*E*)-3-hexenol, and methyl benzoate [27]. Some important variations in fruit aroma volatiles produced by different kiwifruit varieties have been found to be due to the presence or absence of diverse sulfur-containing VOCs.

Mango (*Mangifera indica* L.) fruit aromas contain more than 270 VOCs in different mango varieties [74]. Monoterpenes are the most important compounds contributing to mango flavor [75]. Generally, terpenes are the major class of compounds in New World and Colombian mangoes whereas alcohols, ketones, and esters are mainly responsible for the characteristic aroma of Old World mangoes [76,77].

At least 280 VOCs have been found in pineapple (*Ananas comosus* L. Merr.) fruit aromas [30]. Esters and hydrocarbons were found to be the major constituents of fruit aromas, whereas octenoic acid, methyl ester, hexanoic acid, octanoic acid and ethyl ester were minor aromatic components. The relative content of different volatiles in pineapple aroma varied significantly during fruit development [49].

Raspberry (*Rubus idaeus* x *ursinus*) fruit aromas are composed of at least 200 volatile compounds that vary in concentrations for different cultivars. Many alcohols, aldehydes and ketones (including raspberry ketone, α-ionone, β-ionone, linalool, (*Z*)-3-hexenol, geraniol, nerol, α-terpineol, furaneol, hexanal, β-ocimene, 1-octanol, β-pinene, β-damascenone, ethyl 2-methylpropanoate, (*E*)-2-hexenal, heptanal, and benzaldehyde have been identified in raspberry aroma [13]. Among them, α-ionone, β-ionone, geraniol, nerol, linalool, and raspberry ketone probably contribute most to red raspberry aroma [31].

The complex fruit aroma of strawberry (*Fragaria* x ananassa Duch.) contains approximately 350 volatile compounds [3,78]. The furanone compound (furaneol), 2,5-dimethyl-4-hydroxy-3(2*H*)-furanone, and its methyl derivative (mesifurane) are considered the dominating compounds that contribute the typical caramel-like, sweet, floral and fruity aroma [17]. Aldehydes and alcohols (such as hexanal, *trans*-2-hexenal and *cis*-3-hexen-1-ol) contribute the unripe notes to green strawberry aroma in which the concentrations of these components are cultivar and ripeness dependent [17].

Notice from comparisons of key volatiles (principal components) that distinguish between different fruit types that certain variations in fruit volatiles from specific chemical classes often are most useful for fruit aroma discriminations. For example, apple and pineapple varieties may be distinguished primarily by differences in aroma ester composition, *i.e.*, variations in ester volatiles present in fruit aromas, whereas grapes and mango varieties are discriminated mostly by terpene volatiles that are detected in the aroma. By contrast, banana varieties are distinguished mainly by aldehydes and aliphatic alcohol volatiles, but ketones, furaneols, and alcohols are more important for distinguishing between blackberry and raspberry varieties. Discriminations between blueberry varieties are determined predominantly by the presence of esters, aldehydes, and terpenoids in the fruit aromas.

Despite the fact that a very large number of VOCs have been detected in various fresh fruit types, only a small fraction of these compounds have been identified as contributing significantly to the impact components (top and middle notes) of fruit aroma based on quantitative abundance and human olfactory thresholds [79]. The human olfactory threshold (for a particular aroma) is defined as the lowest concentration of aromatic compounds present in an aroma in which human subjects (usually 50 percent of a human panel) can smell the presence of the aroma [80].

Differences in the aroma characteristics of different fruit varieties are attributed to variations in their chemical profiles, based on the types of VOCs present and the relative concentrations of individual volatiles found in the aroma mixture. The principal VOCs, found in the aromas of specific fruit cultivars, may be used to distinguish between different fruit varieties. These differences in aroma composition and relative abundance of fruit volatiles in different fruit varieties are the means by which e-nose devices are capable of recognizing differences in fruit aromas and discriminating between fruit cultivars based on their distinct aroma signature patterns resulting from variations in e-nose response (sensor-array outputs) to different fruit aromas. Thus, e-nose discriminations of fruit aroma are determined both by the volatiles present and molar ratios of individual components found in each aroma (gaseous mixture)s.

## 3. Electronic-Nose Applications for Fruit Aroma Characterizations

Initial interest in the use of electronic noses as a non-destructive method to study the characteristics of fruits was shown by Benady *et al.* [81] who developed a sensing machine with a single semiconductor gas sensor in 1995, located within a small cup, placed on the surface of fruits of three different muskmelon cultivars. The instrument could discriminate between ripe and unripe fruits with an accuracy of 90.2%, and sort fruits into three ripeness categories (unripe, half-ripe and fully ripe) with an accuracy of 83%. The same research group worked on blueberries (the same year) to determine the variability in e-nose response among blueberry cultivars and to assess ripening stage and fruit quality (Simon *et al.*) [82].

Since these pioneering works, researchers have focused on developing and testing non-destructive sensorial techniques for the evaluation of the many and diverse characteristics and qualities of various fruits, particularly fruit maturity stages, shelf life and genotypic effects on aroma or bouquet characteristics. A comprehensive list of e-nose applications for characterizations and chemical discrimination of fruit volatiles for many fruit types is given in Table 3.

### 3.1. Apples and Pears

The fruit species receiving the most interest among e-nose researchers is apple (*Malus domestica* Borkr). Bai *et al.* [83] examined changes in the aroma profile on freshly-cut Gala apple slices treated with ethanol vapor, heat and 1-methylcyclopropene to prolong visual shelf-life. The FOX 4000 e-nose system (Alpha MOS, Toulouse, France) was utilized to assess aroma quality and the results demonstrated that pretreatments with ethanol and heat are effective in prolonging visual shelf life, but at the expense of aroma quality.

One of the main needs of fruits producers is to determine optimal picking date for different fruit types in order to assure the presence of preferred traits expected by consumers for maximum salability. Harvesting fruits at the optimal physiological condition results in fruits with the highest quality characteristics (aroma, firmness, color, flavor), and an extended shelf life. Fruit picked too early will not ripen sufficiently after storage and may suffer from physiological disorders, whereas those picked too late will be mealy and too soft following storage. Traditional techniques for assessing apple quality are destructive, requiring that only random samples are tested. For these reasons, electronic noses have been preferred for assessing the maturity stage of apples (at harvest) since 1999 to determine the optimal picking date. Hines *et al.* [88] first tested a prototype e-nose equipped with four commercial tin- oxide gas sensors on “Golden Delicious” apples to accurately classify fruit ripeness. The same year, Young *et al.* [98] worked on Gala apples, discriminating the ripening stage at harvest in four different classes. Saevels *et al.* [95] used the Technobiochip (Elba Island, Italy) Lybra Nose for predicting the optimal harvest date of apples as well as the cultivar effect on aroma of “Jonagold” and “Braeburn” varieties. The e-nose used in this study contained a sensor array based on Quartz Crystal Microbalance (QCM) sensors coated with metalloporphyrins and related compounds. Data were collected for two years and the results yielded a good predictive model developed for each cultivar based on one year of data, but a similar model based on two years of data was less effective at predicting optimal harvest dates.

Another commercial electronic nose (Cyranose 320, Cyrano Sciences Inc., Pasadena, CA, USA) was employed by Pathange *et al.* [93] to assess apple maturity for the apple cultivar “Gala”. This instrument could positively classify the fruits into three groups according to their maturity stage (immature, mature and over mature) with an accuracy of 83%. Brezmes *et al.* [85–87] published several works on the assessment of apple ripeness. They initially worked on “Pink Lady” apples using a prototype e-nose equipped with 21 commercial tin oxide or Metal Oxide Semiconducting (MOS) sensors. The accuracy of e-nose classification of fruit maturity stage depended on the statistical classification technique used on e-nose data. PCA analysis did not show any clustering behavior that could be attributed to ripening, whereas a neural network classification algorithm provided good results. Brezmes *et al.* [85] could not determine the correct maturity stage of some apple cultivars, but had very good results on peaches and pears. More recently, Baumgartner *et al.* [84] showed very good results in discriminating the ripening stage of “Golden Delicious” apples, using a lesser-known Swiss e-nose (SMart Nose, SMart Nose SA, Marin-Epanier, Switzerland).

Of all commercial fruit species, apples rank highest with the largest number of experimental and commercial cultivars (including ancient, traditional and modern varieties) available in world markets. Apples have the greatest diversity of flesh and pericarp colors, flesh firmness, shelf life, and especially flavors and aromas. The cultivar effect, or variation in aroma characteristics or bouquet of apples, was studied by Marrazzo *et al.* [92] using a Cyrano Science prototype e-nose equipped with an array of 31 chemical sensors (Pasadena, CA, USA) to discriminate between “McIntosh”, “Delicious” and “Gala” apple varieties. This electronic-nose prototype discriminated between fruit-aroma classes (varieties) based on data from the first day after harvest when the cultivar effect on aroma was more intense for freshly-harvested fruits. A more difficult test was recently performed by Pruteanu *et al.* [94] who successfully employed a FOX 4000 e-nose to discriminate between seven different varieties of Romanian apples.

One of the most important quality features of any fruit species or variety is the duration or longevity that optimal fruit characteristics can be maintained, referred to as shelf life, prior to decline to an unsalable state. The ultimate goal is to develop fruit cultivars with a very low after-harvest perishability coefficient, indicating a low cull-rate following commercial display to consumers. The merchantability of fresh products is largely influenced by shelf life. Traditionally, shelf life is measured and assessed through an evaluation of the chemical and physical properties (factors) that most determine ripening, maturation and post-harvest deterioration. Some of the key parameters measured included color, soluble solids content (SSC), percent of sugar (brix), and titratable acidity (TA). All of these parameters are commonly used by researchers and industry fruit graders, but none of these parameters are utilized by or matched with criteria used in fruit selection from the consumer perspective. Thus, measuring and evaluating post-harvest perishability through one single parameter, changes in volatile compounds directly associated with aroma, is market-oriented. Herrmann *et al.* [89] probably was the first researcher to become interested in monitoring aroma of apples during shelf life. It was well known at that time that the ratio of aldehydes within apple headspace volatiles could be used as indicators of ripeness for many apple varieties. Consequently, a QCM prototype electronic nose containing sensors coated with aldehyde-sensitive materials was used to monitor the increase in *trans*-2-hexenal concentration as an indicator of post-harvest development over time. Saevels *et al.* [96] and Baumgartner *et al.* [84] monitored the post-harvest shelf life as well, obtaining very interesting good results, as did Guohua *et al.* [90] with “Fuji” apples, employing an electronic nose prototype equipped with an array of eight tin oxide MOS sensors.

Li *et al.* [91] examined the aroma bouquet of deteriorating apples during the shelf life period. Physical damage to apples dramatically reduced economic value due to changes in color, shape, flavor and aroma, as well as increased susceptibility to attack by various post-harvest pathogens. Results of this work demonstrate that differences in numbers of physical cuts to the fruits had effects on volatile compound emissions. Apples subjected to two and three cuts generated aroma bouquets significantly different from uncut fruits. A similar study was published by Di Natale *et al.* [129] who used the Technobiochip Lybranose QCM e-nose to successfully discriminate between no cuts, one and two cut fruits. Xiaobo *et al.* [97] utilized a more comprehensive approach by combining three different sensors (a near-infrared spectrophotometer, a machine vision system and an electronic nose) to classify “Fuji” apples according to several quality parameters.

Other researchers have focused on pears (*Pyrus* species) to assess the maturity stage at harvest and shelf-life. Oshita *et al.* [130] successfully classified “La France” pears into three groups according to three storage treatments applied after harvest to prolong the shelf-life by using an Aromascan conducting polymer (CP) e-nose. Zhang *et al.* [131,132], published similar papers on the prediction of acidity, soluble solids content and firmness of “Xuequing” pears by employing a prototype e-nose equipped with eight different MOS sensors and an artificial neural network to analyze their response. Finally, Li *et al.* [164] worked on a Chinese species of *Pyru*s (*Pyrus ussuriensis* Maxim.) to characterize its volatile organic compounds (VOCs) at different ripening stages by traditional methods compared to an electronic nose.

### 3.2. Peach and Apricot

Three research groups have worked on apricot (*Prunus armeniaca* L.), obtaining good results by the use of three different commercial electronic noses [99–101]. The cultivar effect on aroma bouquet was tested using a PEN2 electronic nose, a portable (AlphaMos, Schweirin, Germany) and light-weight sensing machine [100]. It consists of a sensor array composed of 10 different doped semi-conductive MOS sensors positioned in a small chamber. The signal of the electronic nose was statistically analyzed by a trained artificial neural network (ANN) which is a data-processing tool that mimics the structure of the biological neural system, exhibiting brain characteristics of learning. In association with gas chromatography-mass spectroscopy (GC-MS), a FOX 4000 e-nose was used in the second study to characterize and discriminate between eight different apricot cultivars with promising results. Non-destructive cultivar assessment is very important in particular for apricot fruits to classify unknown samples and to prevent adulterations.

Defilippi *et al.* [99] worked on post-harvest quality of apricots, assessed by changes in VOCs detected with an EOS 835 electronic nose (Sacmi scarl, Imola, Italy). In order to determine differences in aroma profile, apricots were harvested at two maturity stages and stored at 0 °C and 20 °C (shelf-life simulation) for 15 and 30 days, then analyzed by a trained panel test and electronic nose. This e-nose could not classify maturity level of cold samples, but could only be classified after simulated shelf-life and panel test.

Peaches, *Prunus persica* (L.) Batsch and *P. persica* var. *nucipersica*, have been thoroughly studied by researchers interested in finding new ways of characterizing fruits in a non-destructive way. The first report come from Brezmes *et al.* [85]. The initial approach was aimed at discriminating between cultivars in an attempt to assess the feasibility of a fast, non-destructive, and cheaper way to classify unknown samples and decrease food frauds [133,135–137,139,140].

Peaches, nectarines and most varieties of *P. persica* are climacteric and particularly perishable at harvest and during storage, requires that these fruits be maintained at 0 °C for only a few days because shelf-life of this species is particularly short. These are only some of the reasons why producers utilize many alternative methods to evaluate the maturity stage of the fruit directly on the tree, traditionally based on personal experience. The same thing is asked by industry and retailers who need non-destructive, fast and systematic methods to evaluate the shelf life of fruits and other perishable products. Most of the previously mentioned researchers, including Brezmes *et al.* [87] and Zhang *et al.* [142], have tried to test the feasibility of using an electronic nose to assess shelf life of fruits. Benedetti *et al.* [133] employed a PEN2 e-nose to successfully classify samples of four different cultivars of peach according to their ripening stage. Performing Principal Components Analysis (PCA) on sensor data, peaches showed a linear data distribution for PC1 (from right to left), with increasing days of shelf-life. They concluded that no more than three sensors had a high influence on the sensor-output pattern for the fruit aroma, but only one sensor was relevant in the discrimination of peaches on the basis of shelf-life. They interpreted this single-sensor response to indicate that the sensor signal was directly linked to ethylene production, responsible for ripeness of peaches. A similar response was published by Rizzolo *et al.* [138] by using a similar but more advanced electronic nose (PEN3) manufactured by the same Company. In this case, only three sensors seemed to be associated with fruit ripeness and a linear correlation between PCs and quality indices indicated PC1 was related to ethylene production as well. More recently, Guohua and colleagues developed a model for the prediction of peach freshness based on a home-made electronic nose [165].

Monitoring the sensorial qualities of stored (refrigerated) fruits, in particular the loss of flavor and aroma of peaches throughout the period between harvest and the arrival at retail stores and during transportation to far-away markets, is one of the main problems facing fresh-fruit exporting companies and producers. Electronic noses were successfully employed by Infante *et al.* [136] and Zhang *et al.* [143] to evaluate the development (or loss) of aroma during transportation to markets. In particular, Infante *et al.* [137] could discriminate between aroma qualities of four cultivars, showing that “Tardibelle” peaches have the highest quality attributes even after 42 days of cold storage following harvest.

Zhang *et al.* [141,142,166] attempted to establish a quality index model to describe the effects of different picking dates of peaches by the use of a self-made prototype e-nose equipped with eight commercial tin semiconductor sensors and a commercial acquisition card. Sensor responses were validated by traditional peach quality parameters such as firmness, sugar content and pH at three different picking times. With the aim of predicting fruit quality based on these parameters, Principal Component Regression (PCR) and Partial Least Squares (PLS) regression where applied. The results showed that the two methods allow the determination of firmness, sugar content and pH by use of electronic nose. A similar research was performed by Su *et al.* [140] who tried to assess harvest season and quality of 39 cultivars of peaches and nectarines. By using a FOX 4000 e-nose and manipulating data by Discriminant Function Analysis (DFA), they successfully linked sensor responses to total soluble solids (TSS) concentration and titratable acidity (TA) in all harvest seasons, but only for those samples with a low or high concentration of TSS and a low or high TSS/TA ratio. Infante *et al.* [137] employed the Italian EOS 835 (Sacmi, Imola) e-nose to predict the quality of four peach cultivars by applying a multiple linear regression (MLR) to sensors-response data. They were able to describe the quality attributes “acidity”, “sweetness” and the more general “acceptability” by the use of the e-nose, concluding that the instrument could discriminate between peach varieties through descriptors that mainly determine acceptability by the peach consumer. A similar paper was published by Di Natale *et al.* [134] who applied both electronic nose and panel sensor analysis to determine through an advanced data analysis, some proper sensorial indicators for the classification of peach fruits according to consumer palatability.

### 3.3. Citrus

Fruits belonging to the complex genus Citrus are commonly called citruses. These fruits are well known since ancient times for their nutraceutical properties, providing medical and health benefits,and the unmistakable pungent notes of their aroma. Hernandez Gomez *et al.* [119,121] worked on mandarin (*C. reticulata* Blanco) by using a PEN2 e-nose to associate sensor responses to harvest date (five different dates = aroma classes). No more than three sensors were employed in the Linear Discriminant Analysis (LDA), which gave clearer results than PCA. The later work by Hernandez Gomez *et al.* [120] was focused on evaluating the change in aroma bouquet emitted by mandarins during different storage treatments (plastic bag, paper box and refrigerator) and time (shelf-life), as well as other quality parameters. In this case, no significant predictive results were shown, while quality indices such as firmness were predicted by e-nose sensor responses.

Di Natale *et al.* [129] conducted research on oranges using a QCM prototype electronic nose. They distinguished between the different storage days (duration) for oranges, whereas Russo *et al.* [104] recently were able to discriminate between genuine bergamot (*C. bergamia* Risso et Poiteau) essential oils from other non-genuine types in order to defend the uniqueness of this very economically-important Southern Italian product. Akakabe *et al.* [149] worked on some Chinese species and varieties of citruses (including *C. nagato-yusukichi* Tanaka, *C. sudachi* Hort. ex Shirai, *C. junos* Siebold. ex Tanaka, *C. sphaerocarpa* Tanaka) to evaluate the capability of an electronic nose to discriminate between species and varieties by their aroma profiles.

### 3.4. Grape

One of the most important and widely-studied applications of electronic gas-sensing machines (electronic noses and tongues) in the food industry concerns analysis of the aroma characteristics of wine, the alcoholic product of fermented grapes [167–173]. However, some researchers have assessed the possibility of employing these gas-sensing machines directly on grapes in the field, or immediately after harvest, to provide important data on fruit quality and physiochemical parameters that are vital to the production success of quality wines and to limit food frauds or adulterations by dilutions with cheaper products. In this regard, Zoecklein *et al.* [114] studied the effects of ethanol treatments on volatile aromatic compounds emitted by two *V. vinifera* L. varieties (“Cabernet Franc” and “Merlot”) at the onset of ripening (veraison). Both a conducting polymer (CP) e-nose and a surface acoustic wave-based (SAW) e-nose were capable of successfully discriminating between treated and untreated fruits.

A very interesting paper was published by Devarajan *et al.* [115] who discovered that the canopy side (north versus south and east versus west) has an effect on the aroma bouquet emitted by fruits (and wine) of Cabernet franc. Data were processed by using two different electronic noses (a Cyranose 320 and a zNose 730) on the basis of two growing seasons. Both sensing systems provided effective discrimination of canopy sides for grapes VOCs using canonical discriminant analysis.

The maturity level at harvest over two seasons was evaluated by Athamneh *et al.* [110] using a portable Cyranose 320 e-nose on “Cabernet Sauvignon” grape samples picked at three different maturity stages. The instrument proved capable of discriminating between different stages of maturity and fruits from different vine canopy sides.

Post-harvest dehydration is one of the most important steps in the wine-making process. Although the volatile fraction of a wine can be formed by hundreds of chemically-different compounds, the aroma compounds formed during drying have significant effects on wine quality. Currently, winemakers judge optimum drying times in terms of sugar concentration (brix) or water loss rather than based on more precise continuous monitoring of aroma profiles by e-noses until optimum drying conditions are met. Thus, some researchers have tried to determine the optimum drying times for wines using an electronic nose to assess the quality of aroma bouquets derived from dehydrated grapes [111–113]. In all cases, very good results were obtained by the use of a QCM prototype electronic nose developed by the University of Rome Tor Vergata.

### 3.5. Strawberry and Other Berries

To our knowledge, only three papers have been published on different applications of electronic noses for the evaluation of aroma characteristics in strawberry (*Fragaria x ananassa* Duch.) fruits. Agulheiro-Santos [151] reported on the capability of an electronic nose to assess the influence of different nitrogen fertilizations on the aroma quality of “Camarosa” strawberry. This represents one of the few examples of scientific research into electronic-nose technologies being applied directly to evaluate and modify growth protocols and agronomic techniques for the improvement of fruit aromatic and flavor qualities.

Strawberry fruit maturity was evaluated using a MOS electronic nose containing 18-metal oxide gas sensors [152]. The instrument discriminated between five stages of fruit maturity (from white to overripe) and three picking dates for two varieties. A PEN2 e-nose was employed by Qiu *et al.* [153] to characterize the aroma of five strawberry varieties, derived from freshly-squeezed juice produced according to a squeezing-processing technique.

Blackberry (*Rubus glaucus* Benth), bilberry (*Vaccinium meridionale* Swartz) and blueberry (*Vaccinium* spp.), comprise a group of fruits called “berries” with peculiarities of shape and size (always round and small fruits), with colors-ranging from yellow to black-blue-and flavor (sweet or sour). This definition of berry is not linked to the botanical meaning of the term “berry”. Berries are among the most perishable fruits before and after harvesting. Thus, they have to be very carefully harvested and processed at the proper time and in specific ways to maintain quality and shelf life. Quality control in this field is particularly important. Simon *et al.* [82] tested a very early e-nose prototype, equipped with only two commercial sensors, to assess the maturity level of harvested fruits based on aromatic profile, and to detect damaged fruits in a closed container. The nutraceutical role of blackberries and bilberries was assessed by Bernal *et al.* [105]. In addition, changes in volatile components during different stages of fruit maturity were evaluated by employing a PEN3 electronic nose [106].

### 3.6. Mango and Other Tropical Fruits

Among so-called “tropical fruits”, mango *(Mangifera indica* L.) is the most studied species in the field of gas-sensing e-nose machines. The first report by Lebrun *et al.* [123] attempted to assess the optimal harvest date. Whole and homogenated mango fruits were sampled and the aroma from each fruit was analyzed using a FOX 4000 e-nose. Although a deep study on the optimal dilution of the sample was carried out, this e-nose was not effective in determining the harvest date. Similar mediocre results were obtained by Kitthawee *et al.* [122], involving a study of hard green mangoes. The electronic nose could correctly classify only 68% of fruits according to their ripening stage, too low to be introduced into the retail market as a non-destructive method to assess ripening stage in mangoes. Afterwards, several researchers pursued the same goal some years later. Lebrun *et al.* [124] worked on three different mango cultivars (‘Cogshall’, ‘Kent’ and ‘Keitt’) harvested at different fruit maturities. This e-nose could separate fruits from different picking dates as well as fruits from different varieties. Zakaria *et al.* [125] published similar good results employing a Cyranose 320 e-nose.

A pioneering work by Llobet *et al.* [102], utilizing a self-made electronic prototype equipped with a tin-oxide MOS commercial sensor array, was able to discriminate between different stages of fruit maturity in banana (*Musa x paradisiaca L*.) fruits, and also predict the maturity stage of unknown samples, applying a neural-net classifier.

The Italian research group of Torri *et al.* [146] worked on pineapple (*Ananas comosus* L. Merr.) fruits. One experiment was aimed at monitoring the freshness of minimally-processed slices of pineapple during storage by use of a PEN2 electronic nose. Results indicated that this e-nose was successfully employed in the field to determine that pineapple fruit is particularly perishable and that even minimally-processed pineapple fruits lose their aroma characteristics very quickly.

Other researchers have evaluated the maturity stage for harvesting tropical fruits. Supriyadi *et al.* [148] employed a FOX 4000 e-nose to discriminate between ripe and unripe snake fruits (*Salacca edulis* Reinw.). Pokhum *et al.* [109] identified the ripeness stage of durian fruits (*Durio* spp.) by use of a MOS e-nose. Márquez Cardozo *et al.* [150] worked on the Columbian exotic and highly perishable soursop fruit (*Annona muricata* L.) a member of the custard apple tree family (Anonaceae). In this case, a PEN3 e-nose easily classified fruit samples as unripe, half ripe, ripe or overripe. Nugroho *et al.* [147] recently utilized an array of four commercial tin-semiconductor sensors, their prototype electronic nose, to discriminate between different maturity-classes sapodilla (*Manilkara zapota* (L.) P. Royen) fruits.

### 3.7. Other Miscellaneous Fruits

A paper by Lebrun *et al.* [108] hitherto has been the only research study to report on the application of an electronic nose for the rapid and non-destructive discrimination of date (*Phoenix dactylifera* L.) varieties. The research group of Alasalvar worked successfully on five raw and eighteen roasted Turkish hazelnuts (*Corylus avellana* L.) in an attempt at characterizing differences in aroma bouquet according to variety [116,117].

Garcia-Breijo *et al.* [144] and Li *et al.* [145] investigated persimmon (*Diospyros kaki* L.) fruits to discriminate between two different cultivars using a semiconductor commercial e-nose sensor array to assess fruit ripening stage and storage life, applying PCA and LDA statistical methods to a PEN3 e-nose sensor output data. They also attempted to determine the fewest number of sensors that could explain all the variance. Guarrasi *et al.* [118,174] worked on loquats [*Eriobotrya japonica* (Thunb.) Lindl.], a plant belonging to the Rosaceae family native to Japan and China and also wide spread in the Mediterranean Regions of Italy. The fruits were characterized chemically (SSC, TA, pH), morphologically, electronically using an e-nose, and olfactorily (via a trained human panel test). Although cross-data from traditional instrumental techniques and e-nose could identify aroma features, neither the panel test nor the electronic nose could discriminate between the four different sample cultivars.

Very recently, a paper by Ghouhua *et al.* [175] reported about a quality forecasting method using a home-made electronic nose based on an eight MOS sensors array. Samples of Winter jujube (*Ziziphus jujuba* Mill.) were analyzed each day, for 8 days, for physical and chemical indexes as well as via EN. PCA results indicated that jujubes under different time had an approximate trend, but the samples could not be qualitative or quantitatively discriminated from each other.

### 3.8. Vegetable Fruits

From a botanic point of view, tomatoes and other vegetables are actually fruits because they are derived from the ripened ovary of a flower [176]. Nevertheless, the general public views these fruits as vegetables especially from a culinary point of view. Quality attributes like the perfect maturity stage at harvest, long shelf-life and attractive visual appearance are critical factors that must be taken into account when evaluating agricultural protocols. Within all vegetable-type fruits, tomato (*Solanum lycopersicon* L.) is the species receiving the most attention in scientific research efforts in the field of electronic sensing since 1997. Maul *et al.* [160,161] published some early results on the evaluation of an electronic nose to identify and discriminate tomatoes, exposed to different harvesting and postharvest handling treatments, in order to measure quality diversities (in flavor, aroma and other quality parameters) between physiological (portable) maturity and market maturity, highly influenced by long-distance handling (during transportation) and even more by marketing systems.

Sinesio *et al.* [162] employed a prototype electronic nose and a panel test to discriminate between fruit samples based on different qualities of tomatoes. Fruits were harvested from two different Italian farms (one of them conducting traditional agriculture, the other organic farming) and classified as “very good”, “good”, “fair” and “poor”, according to visual selection for the presence of injuries and physical damage. A QCM electronic nose was used, containing eight sensors coated with different metalloporphyrines. Their data showed that through the use of a neural-net statistical algorithm, the e-nose could discriminate between classes better than a trained human panel test.

Electronic noses can actually discriminate between different levels of mechanical damage in fruits like tomatoes which apparently release different VOCs according to different degrees of damage [156]. Bruising fruits usually lead to enhanced ripening at a rate proportion to the amount of damage.

Shelf life of tomatoes was assessed using electronic noses by Berna *et al.* [154,155] and by Hernandez Gomez *et al.* [158] in 2008. In both cases utilizing three different e-noses (a Technobiochip Lybranose, a University of Rome “Tor Vergata” enQbe, and a AlphaMos PEN2), a clear separation of different aroma classes was not achieved, whereas very good results were obtained by Hernandez Gomez *et al.* [157] in discriminating between different fruit maturity stages at harvest. Wang and Zhou [163] also obtained good results by crossing PCA sensor data with firmness data.

Hong and Wang [159] recently found that it was relatively simple to identify high quality fruits visually at harvest, but it is very difficult to assess the freshness of squeezed cherry tomatoes (in 100% tomato juices) unless traditional and time-consuming instruments are employed. Unfortunately, their electronic nose system did not give very convincing results.

Abbey *et al.* [126,127] and Russo *et al.* [128] worked on bulbs of *Allium* ssp. which are not named “fruits” either from a botanical or culinary point of view. We report these studies here because these species are somehow used as “fleshy” vegetables in kitchens. The initial work on *Allium* species was aimed at assessing the ability of an electronic nose to discriminate between different species (*A. sativum* L., *A. ampeloprasum* var. *porrum, A. cepa* var. *aggregatum*, and *A. cepa* L.) on the basis of their aroma profile. Based on pyruvic acid and thiosulphinates content (which characterize these species and varieties), the e-nose could successfully discriminate between them. In a secondary experiment, an electronic nose was employed to evaluate the effects of some agronomic factors, such as fertilizations with nitrogen (N) and sulfur (S) as well as the soil effects on plant aroma characteristics in the field and greenhouse. This is an interesting application of an e-nose instrument devoted to classifying different aroma bouquets in agricultural products whose final quality is so influenced by aroma profiles, such as for white pepper (*Piper nigrum* L.). Mamatha and Prakash [177] could easily discriminate between three cultivars of pepper, whereas a recent work of Liu *et al.* [143] was devoted to examining flavor quality of five new pepper genotypes. In all cases, the α-Gemini (Alpha MOS SA, Toulouse, France) e-nose, equipped with an array of 6 MOS sensors that can be chosen and customized by users, was useful in identifying known and unknown samples. Another example of this application in which an e-nose was used to identify unknown genotypes of fruit vegetables was published by Zawirska-Wojtasiak *et al.* [107]. Theyattempted to use the electronic nose to differentiate between transgenic lines of cucumber (*Cucumis sativus* L.) compared to controls.

The protection of specific geographical labels (as sources of fruits) from frauds and adulterations is one of the main concerns of producers, industry and final users as well as those who want to be extremely sure about the quality and the exact origin of fruits used in their businesses. Italy has the highest number of European protected denominations concerning fresh products [103]. An electronic nose was used to identify the geographical origins of two local varieties of bell pepper (*Capsicum annuum* L.) and this e-nose was applied in association with other traditional methods to characterize the product according to morphometric, qualitative, spectroscopic and aromatic data.

## 4. Conclusions

Electronic-nose devices have been utilized in a wide range of diverse applications in the agriculture and forestry industries to improve the effectiveness, efficiency and safety of processes involved in the production of quality food and fiber plant-based products [5]. As summarized in this review, e-nose instruments also offer many new potential applications for the fruit-production industry to facilitate many tasks involving fruit aroma evaluations during all stages of the agro-fruit production process from early cultivation activities, field-applied pest control applications, and timing of fruit harvests to many post-harvest stages including fruit transportation, storage, and finally the maintenance of fruit shelf life during display in commercial markets.

The potential for future developments and new aroma-based applications of electronic-nose devices in fruit production processes include e-nose detection of pesticide residues on harvested fruit surfaces to facilitate enforcement of human health regulations of the Environmental Protection Agency (EPA) [178–180], post-harvest fruit disease detection and management [5,8], and monitoring gases released from fruits in storage to control fruit ripening (maintain fruit shelf life) and fruit quality.

## Figures and Tables

**Table 1.** Chemical classes of VOCs that are principal components of distinctive fruit aromas.

**Fruit Type**	**Chemical Class**	**Example Compounds**	**Chemical Structure**	**Reference**
Apple	Aliphatic esters	ethyl butanoate	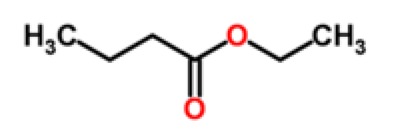	[18–20]
Apricots	Aliphatic alcohols	1-hexanol	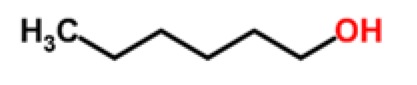	[21,22]
Banana	Aliphatic esters	isoamyl acetate	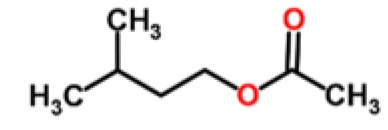	[23]
Caraway	Terpenoids	carvone	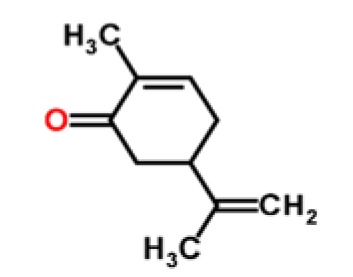	[24,25]
Cantaloupe	Thiobutyrates	S-methyl thiobutanoate	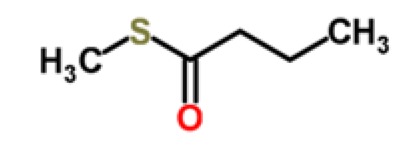	[26]
Kiwifruit	Aliphatic aldehydes	hexanal	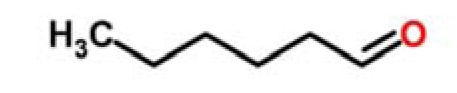	[27]
Peach	Lactones	γ-decalactone	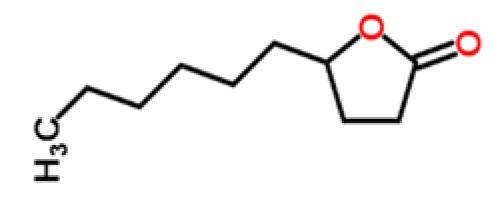	[28]
Peach	Aliphatic esters	hexyl acetate	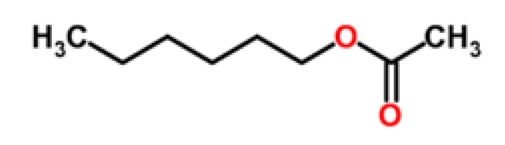	[29]
Pineapple	Organic acids	hexanoic acid	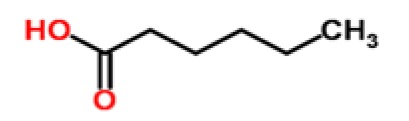	[30]
Raspberry	Aliphatic ketones	raspberry ketone	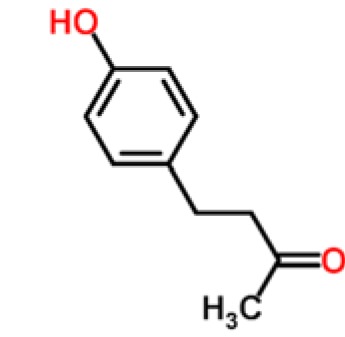	[31,32]
Strawberry	Furanone lactones	furaneol	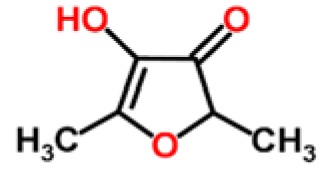	[17]
Tomato	Apocarotenoids	β-ionone	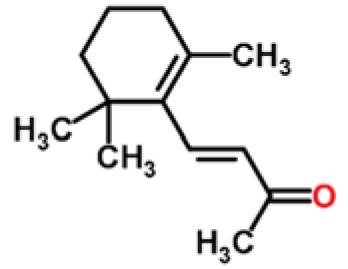	[33]

**Table 2.** Principal volatile compounds comprising the distinctive aromas of different fruit cultivars.

**Fruit Type**	**Cultivars/Varieties**	**Principal Volatile Compounds in Aroma ^†^**	**References**
Apple	Cox orange	Acetaldehyde, ethyl butanoate, ethyl methyl propanoate, 2-methyl butanol	[18]
	Elstar	Ethyl butanoate, ethyl 2-methyl butanoate	[18]
	Fuji	Ethyl 2-methyl butanoate, 2-methyl butyl acetate, hexyl acetate	[18]
	Pink Lady	Butyl acetate, hexyl acetate, 2-methylbutyl acetate, hexyl butanoate, hexyl 2-methyl butanoate, hexyl hexanoate	[20]
Banana	Cavendish	(*E*)-2-hexenal, acetoin	[38]
	Frayssinette	2, 3-Butanediol, solerol	[39]
	Plantain	(*E*)-2-hexenal, hexanal	[23,38,40]
Blackberry	Black Diamond	Furaneol, 2-heptanol, β-ionone, linalool	[41,42]
	Marion	Furaneol, hexanal, β-ionone, linalool	[41,42]
Blueberries	Primadonna, Jewel	Many aliphatic esters, C_6_-aldehydes	[43]
	Snowchaser, FL02-40	Primarily terpenoids, less aliphatic esters	[43]
Grape	Cabernet Sauvignon	Benzene derivatives, monoterpenes, and sesquiterpenes, (also primarily alcohols)	[44–46]
	Muscat	Citral, citronellol, diendiol I, diendiol II, geraniol, linalool, rose oxide, nerol	[44–46]
	Riesling	Geraniol, α-muurolene, (also primarily esters and aldehydes)	[44–46]
Mango (Columbian)	Haden Irwin, Manila	δ-3-Carene	[47]
	Hilacha, Vallenato	α-Pinene	[47]
	Van Dyke	α-Phellandrene	[47]
	Yulima	Terpinolene	[47]
Pineapple	Cayenne	Ethyl 2-methylbutanoate, ethyl hexanoate, 2, 5-dimethyl-4-hydroxy-3(2*H*)-furanone (DMHF), decanal, ethyl 3-(methylthio) propionate, ethyl butanoate, (*E*)-3-ethyl hexenoate	[48]
	Tainong No. 4	Furaneol, 3-(methylthio) propanoic acid methyl ester, 3-(methylthio) propanoic acid ethyl ester, δ-octalactone	[49]
	Tainong No. 6	Ethyl-2-methylbutyrate, methyl-2-methylbutyrate, 3-(methylthio) propanoic acid ethyl ester, ethyl hexanoate, decanal	[50]

† Principal chemicals (VOCs) found in complex fruit volatile mixtures are listed in alphabetical order, not in order of relative abundance by quantity within mixtures analyzed from individual fruit cultivars or varieties.

**Table 3.** Applications of electronic-nose devices for fruit aroma characterizations.

**Common Name**	**Scientific Name**	**Family**	**E-Nose Type ^†^**	**Discrimination Type**	**Reference**	
Apple	*Malus domestica* Borkr	Rosaceae	FOX 4000 ^1^	Post-harvest treatments	[83]	
			Smart Nose ^2^	Shelf life	[84]	
			Prototype MOS ^3^	Maturity stage at harvest	[85]	
			Prototype MOS	Shelf life	[86]	
			Prototype MOS	Shelf life	[87]	
			Prototype MOS	Shelf life	[88]	
			Prototype QMBs ^4^	Shelf life	[89]	
			Prototype MOS	Prediction of storage time	[90]	
			Cyranose 320 ^5^	Aroma profile during deteriorative shelf life	[91]	
			Unspecified	Cultivar effect	[92]	
			Cyranose 320	Maturity stage at harvest	[93]	
			FOX 4000	Cultivar effect	[94]	
			Libra Nose ^6^	Maturity stage at harvest	[95]	
			Libra Nose	Shelf life	[96]	
			Unspecified	Quality assessment	[97]	
			FOX 4000	Maturity stage at harvest	[98]	
Apricot	*Prunus armeniaca L.*	Rosaceae	EOS835 ^7^	Ripening stage after harvest	[99]	
			PEN2 ^8^	Cultivar effect	[100]	
			FOX 4000	Cultivar effect	[101]	
Banana	*Musa x paradisiaca L.*	Musaceae	Prototype MOS	Ripening stage after harvest	[102]	
Bell pepper	*Capsicum annuum* L.	Solanaceae	Unspecified	Quality assessment	[103]	
Bergamot	*Citrus bergamia* Risso and Poiteau	Rutaceae	ISE Nose 2000 ^9^	Cultivar effect; geographic effect; adulteration	[104]	
Blackberry	*Rubus glaucus* Benth	Rosaceae	PEN3 ^6^	Maturity stage at harvest	[105]	
			Unspecified	Maturity stage at harvest	[106]	
Bilberry	*Vaccinium meridionale* Swartz	Ericaceae	PEN3	Maturity stage at harvest	[105]	
Blueberry	*Vaccinium* spp.	Ericaceae	Prototype MOS	Ripening stage after harvest; quality control	[82]	
Cucumber	*Cucumis sativus* L.	Cucurbitaceae	FOX 4000	Genotypic effect	[107]	
Date	*Phoenix dactylifera* L.	Arecaceae	FOX 4000	Cultivar effect	[108]	
Durian	*Durio* spp.	Malvaceae	Unspecified	Maturity stage at harvest	[109]	
Grape	*Vitis vinifera* L.	Vitaceae	Cyranose 320	Maturity stage at harvest	[110]	
			enQbe ^10^	Dehydration time	[111]	
			enQbe	Dehydration time	[112]	
			enQbe	Dehydration time	[113]	
			enQbe	Post-harvest treatments	[114]	
			zNose ^11^	Canopy side effect	[115]	
			Cyranose 320			
Hazelnut	*Corylus avellana* L.	Betulaceae	E-Nose 4000 ^12^	Cultivar effect	[116]	
			Moses II ^13^	Cultivar effect	[117]	
Loquat	*Eriobotrya japonica* (Thunb.) Lindl.	Rosaceae	Unspecified	Cultivar effect	[118]	
Mandarin	*Citrus reticulate* Blanco	Rutaceae	PEN2	Maturity stage at harvest	[119]	
			PEN2	Post-harvest treatments	[120]	
			PEN2	Maturity stage at harvest	[121]	
Mango	*Mangifera indica* L.	Anacardiaceae	Unspecified	Maturity stage at harvest	[122]	
			FOX 4000	Cultivar effect, post-harvest treatments	[123]	
			FOX 4000	Cultivar effect, maturity stage at harvest, shelf life	[124]	
			Cyranose 320	Maturity stage at harvest	[125]	
Muskmelon	*Cucumis melo* L.	Cucurbitaceae	Not specified	Maturity stage at harvest	[81]	
Onion, spring onion garlic, shallot, leek	*Allium* spp.	Liliaceae	Aromascan CP ^14^	Species effect	[126]	
Onion	*Allium cepa* L.	Liliaceae	Aromascan	Fertilization, soil type effect	[127]	
			ISENose 2000 ^15^	Ecotype effect	[128]	
Orange	*Citrus x sinensis (L.) Osbeck*	Rutaceae	Libra Nose	Shelf life	[129]	
Pear	*Pyrus communis* L.	Rosaceae	Prototype MOS	Maturity stage at harvest	[85]	
			Prototype MOS	Shelf life	[87]	
			Aromascan	Maturity stage at harvest	[130]	
			Prototype MOS	Maturity stage at harvest; quality assessment	[131]	
			Prototype MOS	Maturity stage at harvest; quality assessment	[132]	
Peach	*Prunus persica* (L.) Batsch	Rosaceae	PEN2	Shelf life and cultivar effect	[133]	
			Prototype MOS	Maturity stage at harvest	[85]	
			Libra Nose	Sensorial assessment	[134]	
			Libra Nose	Cultivar effect, quality assessment	[135]	
			Prototype MOS	Shelf life, quality assessment	[87]	
			EOS 835	Cultivar effect, shelf life	[136]	
			EOS 835	Cultivar effect	[137]	
			PEN3	Maturity stage at harvest	[138]	
			FOX 4000	Cultivar effect	[139]	
			FOX 4000	Prediction of harvest time, quality assessment	[140]	
			Prototype MOS	Prediction of harvest time, quality assessment	[141]	
			Prototype MOS	Prediction of harvest time, quality assessment	[142]	
			FOX 4000	Shelf life	[61]	
Pepper	*Piper nigrum* L.	Piperaceae	Alpha Gemini	Genotype effect	[143]	
Persimmon	*Diospirus Kaki* L.f.	Ebenaceae	Prototype MOS	Cultivar effect	[144]	
		Ebenace	PEN3	Maturity stage at harvest and shelf life	[145]	
Pineapple	*Ananas comosus* (L.) Merr.	Bromeliaceae	PEN2	Shelf life	[146]	
Sapodilla	*Manilkara zapota (L.)P. Royen*	Sapotaceae	Prototype MOS	Maturity stage at harvest	[147]	
Snake fruit	*Salacca edulis* Reinw.	Arecaceae	FOX 4000	Maturity stage at harvest	[148]	
Sour citrus	*Citrus nagato*-yuzukichi Tanaka	Rutaceae	Unspecified	Species effect	[149]	
Soursoup	*Annona muricata* L.	Annonaceae	PEN3	Maturity stage at harvest	[150]	
Strawberry	*Fragaria x ananassa* Duch.	Rosaceae	Unspecified	Fertilizations effect	[151]	
			PEN2	Processing approaches effect	[152]	
			PEN2	Processing approaches effect	[153]	
Tomato	*Solanum lycopersicon* L.	Solanaceae	Unspecified	Cultivar effect, shelf life	[154]	
			Libra Nose	Cultivar effect, shelf life	[155]	
			Unspecified	Mechanical deterioration effect	[156]	
			PEN2	Maturity stage at harvest	[157]	
			PEN2	Post-harvest treatments effect	[158]	
Cherry tomato	*Lycopersicon esculentum* var. *cerasiforme*	Solanaceae	PEN2	Shelf life of processed fruits	[159]	
Tomato	*Solanum lycopersicon* L.	Solanaceae	e-Nose 4000 ^12^	Harvesting and postharvest handling treatments effect	[160]	
			e-Nose 4000	Post-harvest treatments	[161]	
			enQbe	Cultivation techniques effect	[162]	
			Unspecified	Maturity stage at harvest and shelf life	[163]	
Ussurian pear	*Pyrus ussuriensis* Maxim.	Rosaceae	Unspecified	Maturity stage at harvest	[164]	

† Electronic nose (e-nose) names, types and manufacturers: 1 = Alpha MOS, Toulouse, France; 2 = SmartNose BV, Amersfoort, The Netherlands; 3 = Self-made prototype equipped with an array of commercial MOS gas sensors; 4 = Quartz microbalances (QMB) gas sensors; 5 = Cyrano Sciences Inc., Pasadena, CA, USA; 6 = Technobiochip, Marciana, Italy; 7 = Sacmi Imola s.c.a.r.l., Imola, Italy; 8 = Airsense Analytics GmbH, Schwerin, Germany; 9 = ISE, Pisa, Italia; 10 = University of Rome ‘Tor Vergata’, Rome, Italy; 11 = Electronic Sensor Technology, Newbury Park, CA, USA; 12 = EEV Inc., Amsford, NJ, USA; 13 = Lennartz Electronic GmbH, Tübingen, Germany; 14 = Osmetech Inc., Wobum, MA, USA; 15 = Labservice Analytica, Bologna, Italy. Unspecified e-nose types were not determinable from descriptions given in the methods section.
